# Refeeding syndrome – awareness, prevention and management

**DOI:** 10.1186/1758-3284-1-4

**Published:** 2009-01-26

**Authors:** Hisham Mehanna, Paul C Nankivell, Jamil Moledina, Jane Travis

**Affiliations:** 1 Institute of Head and Neck Studies and Education (InHANSE), University Hospitals Coventry and Warwickshire, Coventry, UK; 2 Department of ENT, University Hospitals Coventry and Warwickshire, Coventry, UK; 3 Department of Dietetics, University Hospitals Coventry and Warwickshire, Coventry, UK

## Abstract

**Background:**

Refeeding syndrome is an important, yet commonly overlooked condition affecting patients. It occurs when feeding is commenced after a period of starvation. Head and neck cancer patients are at particular risk owing to prolonged periods of poor nutritional intake. This may be from general effects such as cancer anorexia or from more specific problems of dysphagia associated with this group of patients. Awareness of the condition is crucial in identifying patients at risk and taking measures to prevent its occurrence.

**Objectives:**

The aims of this review are to:

1) Highlight the condition and stress the importance of its consideration when admitting head and neck cancer patients.

2) Discuss the pathophysiology behind refeeding syndrome.

3) Review the literature for the best available evidence and guidelines.

4) Highlight the need for further high quality research.

**Conclusion:**

Refeeding syndrome is potentially fatal, yet is preventable. Awareness and identification of at-risk patients is crucial to improving management.

Refeeding syndrome is caused by rapid refeeding after a period of under-nutrition, characterised by hypophosphataemia, electrolyte shifts and has metabolic and clinical complications.

High risk patients include the chronically under-nourished and those with little intake for greater than 10 days. Patients with dysphagia are at particular risk.

Refeeding should commence at 10 kcal/kg per day in patients at risk, and increased slowly. Thiamine, vitamin B complex and multi-vitamin supplements should be started with refeeding.

New NICE guidelines state that pre-feeding correction of electrolyte and fluid deficits is unnecessary, but should be done concurrently with re-feeding.

More research in this field is needed as the evidence base is lacking.

## Background

Refeeding syndrome was first described after the Second World War in prisoners who exhibited cardiac and neurological abnormalities with commencement of feeding after long periods of starvation [1]. Refeeding syndrome is defined as severe, (and potentially fatal) electrolyte and fluid shifts associated with metabolic abnormalities in malnourished patients undergoing refeeding, whether orally, enterally, or parenterally [2]. The hallmark feature is hypophosphataemia, however other biochemical abnormalities are common including disorder of sodium and fluid balance, changes in glucose, protein and fat metabolism, thiamine deficiency, hypokalaemia and hypomagnesaemia [2,3]. It is often not recognised, especially in non-specialist wards.

The incidence of refeeding syndrome is unknown, largely because no universally accepted definition exists. Using a proxy marker to show patients at risk of refeeding syndrome, namely those with severe hypophosphataemia, one study demonstrated a rate of 0.43% in hospital patients, with malnutrition being one of the strongest risk factors [4]. In a well-designed prospective cohort study of a heterogeneous group of intensive care unit ICU patients, 34% of patients experienced hypophosphataemia soon after feeding was started (mean1.9 SD 1.1 days) [5]. Other prospective and retrospective cohort studies of hyperalimentation in ICU have also demonstrated the incidence of refeeding syndrome [2,6].

## Pathophysiology

### Prolonged fasting

In early starvation, blood glucose levels decline, resulting in a decrease in insulin and an increase in glucagon levels. This stimulates glycogenolysis in the liver and lipolysis of triacetylglycerol in fat reserves producing fatty acids (FAs) and glycerol which are used by tissues for energy and converted to ketone bodies in the liver. As glycogen reserves then become depleted, gluconeogenesis is stimulated in the liver, utilising amino acids (derived from the breakdown of muscle), lactate and glycerol resulting in the synthesis of glucose for use by the brain and red blood cells. The main result of these changes is that the body switches the main energy course from carbohydrate to protein and fat. The basal metabolic rate decreases by as much as 20–25% [7].

As fasting continues, the body aims to conserve muscle and protein. The tissues decrease their use of ketone bodies, and use fatty acids as their main energy source. This results in an increase in blood levels of ketone bodies, stimulating the brain to switch from glucose to ketone bodies as its main energy source. The liver decreases its rate of gluconeogenesis due to the reduced need for glucose by the brain thus preserving muscle protein which is its source of amino acids. As a consequence, several intracellular minerals become severely depleted. The concentrations of these minerals (including phosphate) may remain normal in the serum however.

### Re-feeding

The underlying causative factor of refeeding syndrome is the metabolic and hormonal changes caused by rapid refeeding, whether enteral or parenteral. On refeeding, the absorbed glucose leads to increased blood glucose levels, which increase insulin and decrease glucagon secretion. The net result of these changes is the synthesis of glycogen, fat and protein. This anabolic state requires minerals such as phosphate and magnesium and cofactors such as thiamine. Insulin stimulates the absorption of potassium into the cells (via the Na-K ATPase symporter), with both magnesium and phosphate also taken up. Water is drawn in to the intracellular compartment by osmosis. This decreases serum levels of phosphate, potassium and magnesium further, and results in the clinical features of refeeding syndrome.

### Key elements and minerals

#### Phosphorus

Phosphorus is a predominantly intracellular mineral. It is essential for almost all intracellular processes and structural integrity of the cell membrane. It is necessary for energy storage – adenosine triphosphate (ATP), for enzyme/and second messenger activation by phosphate binding, for control of the affinity of the oxygen binding to haemoglobin (via 2,3-diphosphoglycerate[[2,3] DPG], ATP). It is particularly important in the regulation of pH by acid-base buffering.

In refeeding syndrome, long-term depletion of phosphorus in the body occurs along with a greatly increased use of phosphate in the cells caused by the insulin surge. This leads to a deficit in intracellular and extracellular phosphorus. In this environment, even small drops in serum phosphorus may lead to widespread dysfunction of the cellular processes described above [8].

#### Potassium

Potassium is the main intracellular cation. It too is depleted in under-nutrition, whilst its serum concentration usually remains within the normal range. On refeeding, insulin causes potassium to be taken into the cells. This causes significant hypokalaemia and as a result, derangements in the electrochemical membrane potential, potentially leading to abnormalities in cardiac rhythm and even cardiac arrest.

#### Magnesium

Magnesium is an important intracellular ion. It is an essential cofactor in most enzyme systems including oxidative phosphorylation and ATP production. It is also necessary for the structural integrity of DNA, RNA and ribosomes. In addition it affects membrane potential, and deficiency can lead to cardiac dysfunction and neuromuscular complications [9]. Magnesium and potassium levels are linked [10], and so severe hypomagnesaemia will lead to hypokalaemia. Therefore only replacing potassium will not lead to a correction of the potassium deficit, as magnesium replacement has to occur concurrently.

#### Glucose

After starvation, glucose intake suppresses gluconeogenesis by leading to the release of insulin and the suppression of glycogen. If taken in large quantities, glucose intake may therefore lead to hyperglycaemia, with osmotic diuresis, dehydration, metabolic acidosis and ketoacidosis. Excess glucose also leads to lipogenesis (again caused by insulin stimulation). This may cause fatty liver, increased CO2 production, hypercapnoea and respiratory failure [11].

#### Vitamin deficiency

Starvation will usually result in several vitamin deficiencies. The most important of these with respect to refeeding is thiamine, as it is an essential coenzyme in carbohydrate metabolism. Deficiency in thiamine can lead to Korsakoff's syndrome (retrograde and anterograde amnesia, confabulation) and Wernicke's encephalopathy (ocular abnormalities, ataxia, confusional state, hypothermia, coma) [12].

#### Sodium, nitrogen and fluid

Intake of carbohydrate leads to rapid decrease in renal excretion of sodium and water [13]. If fluids are then given to maintain a normal urine output, patients may rapidly become fluid overloaded. This is made worse by the loss of cardiac muscle during starvation. This can lead to cardiac myopathy and reduced contractility further – resulting in cardiac failure.

### Management

#### Early identification of at-risk patients

It is crucially important when considering the management of this condition to ensure clinicians have a clear appreciation of those patients at risk of potential problems, as early detection and prevention of refeeding syndrome is entirely possible. Patients who fall in to this high risk group are summarised in table 1. It is obvious from this table that head and neck cancer patients are at particular risk of this condition, as many have not one, but multiple risk factors. These include periods of poor nutritional intake of more than 5 days for example through inability secondary to the primary tumour causing dysphagia, or in patients who have dysphagia due to strokes. Other head and neck patients at risk include chronic alcohol abuse and high metabolic demands through cancer cachexia or post operative effects of surgery.

**Table 1:** Patients at high risk [2,13,14]

• Oncology patients
• Chronic Alcoholism

• Postoperative patients

• Anorexia Nervosa

• Elderly patients (comorbidities, decreased physiological reserve)

• Uncontrolled Diabetes Mellitus (electrolyte depletion, diuresis)

• Chronic Malnutrition
- Marasmus
- Prolonged fasting/hypocaloric feeding
- Morbid obesity with profound weight loss
- High stress patient unfed for >7 days
- Malabsorptive syndrome e.g. inflammatory bowel disease, chronic pancreatitis, cystic fibrosis, short bowel syndrome

• Chronic antacid users (Mg/Al salts bind phosphate)

• Chronic diuretic users (loss of electrolytes)

#### Nutritional assessment

If suspected, patients should undergo a formal assessment, including a full history focusing on a detailed nutritional intake, alcohol usage, and recent weight change as recommended by the recent NICE guidelines [14] (table 2). It is also important to obtain specialist input from the dietetics department at an early stage. Along with the clinical history, biochemical assessment in the form of baseline phosphate, magnesium, potassium, and sodium blood levels are essential. Micronutrients such as zinc can be assessed at the same time, along with a glucose measurement and renal function.

**Table 2:** NICE Criteria for determining people at high risk of developing refeeding problems [13]

Patient has one or more of the following:
- BMI less than 16 kg/m2
- unintentional weight loss greater than 15% within the last 3–6 months
- little or no nutritional intake for more than 10 days
- low levels of potassium, phosphate or magnesium prior to feeding.
Or patient has two or more of the following:
- BMI less than 18.5 kg/m2
- unintentional weight loss greater than 10% within the last 3–6 months
- little or no nutritional intake for more than 5 days
- a history of alcohol abuse or drugs including insulin, chemotherapy, antacids or diuretics.

#### Treatment of refeeding syndrome

The re-introduction of feeding needs to be approached with caution. Previous guidelines have stressed the importance of adequate replacement of electrolytes, vitamins and minerals before the commencement of feeding, be that enterally or parenterally [15]. This potentially risks prolonging the period of malnutrition the patient has to endure. The newer guidelines from NICE [14] now suggest that as long as replacement occurs in parallel with feeding, this is sufficient.

Vitamin replacement should be started straight away, in particular thiamine and vitamin B to reduce the incidence of Wernicke's encephalopathy or Korsakoff's syndrome, with 200–300 mg oral thiamine daily, and 1–2 tablets vitamin B high potency 3 times daily, and multivitamin or trace element supplement once daily. This replacement once started should be continued for at least 10 days.

If levels of key electrolytes are found to be low, they can be replaced via oral, enteral or intravenous routes depending on how low the levels are and what methods of refeeding are possible. There is little good quality evidence on the best replacement regimes, (one of the key areas where future research needs to be focused) but NICE have made recommendations, including potassium (2–4 mmol/kg/day), phosphate (0.3–0.6 mmol/kg/day), and magnesium (0.2 mmol/kg/day intravenously or 0.4 mmol/kg/day orally).

The rate of refeeding from these same guidelines [14] depends on the severity of the malnutrition prior to refeeding. In moderate risk patients, in any patient who has eaten little or nothing for more than 5 days, the recommendation is a rate of no more than 50% of the energy requirements. If after careful monitoring of clinical and biochemical status, all remains well this rate can start to be increased. If the patient falls in to one of the high risk categories, (see above and tables 1 and 2) replacement of energy should be started slowly with a maximum rate of10 kcal/kg every 24 hours. It can then be increased to meet or exceed full needs over the next 4 to 7 days, and as before, particular attention needs to be paid to biochemical indices and fluid balance. In patients who are very malnourished (body mass index ≤ 14 or a negligible intake for two weeks or more), the NICE guidelines recommend that refeeding should start at a maximum of 5 kcal/kg/24 hours, with cardiac monitoring owing to the risk of cardiac arrhythmias. Circulatory volume should also be replaced but care should be taken not to overload patients.

**Figure 1:**
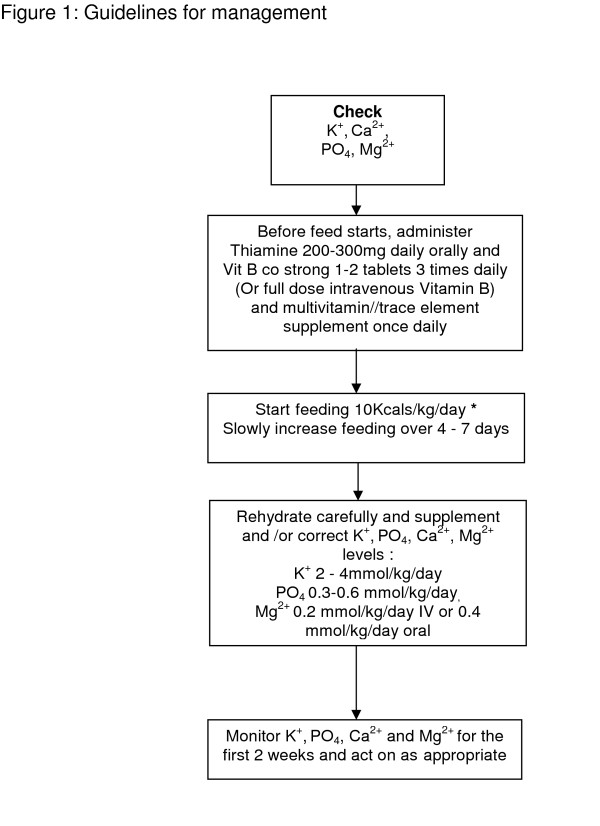
**Guidelines for management**. * if severely malnourished, e.g. BMI less than 14 kg/m or negligible intake for 2 weeks or more, start feeding at maximum of 5 kcal/kg/day. Adapted from NICE [14] and British Association of Parenteral and Enteral Nutrition guidelines [15].

Patients should have daily electrolyte levels checked daily for the first week, followed by three times in the second week. Assessment of urinary electrolytes can be helpful in assessing losses. A summary of the guidelines for management is given in figure 1.

## Conclusion

Refeeding syndrome is an important condition and one that is often diagnosed late in patients at risk. It is particularly relevant to the patients under the care of head and neck surgeons. The key to better patient care in this area is prevention by increased clinician awareness and involvement of specialist dietetic support early on. If patients are diagnosed or suspected then there are now guidelines in place to help with management, however, it must be stressed that many of the recommendations are not based on high quality evidence and this must be highlighted as an area that needs future research time dedicated to it.

## Competing interests

The authors declare that they have no competing interests.

## Authors' contributions

HMM planned the article did the searches and wrote the manuscript. PCN evaluated the evidence, co-wrote and reviewed the manuscript. JM did the searches, and evaluated the evidence. JT did the searches, evaluated the evidence, and reviewed the manuscript.

## About the authors

Hisham Mehanna BMedSc (Hons) MB ChB (Hons) FRCS (ORL_HNS)

Consultant and Honorary Associate Professor

Paul C Nankivell BA BM BCh MRCS

Specialist Registrar

Jamil Moledina BSc (Hons) MBBS MRCS

Senior House Officer

Jane Travis

Macmillan Specialist Dietitian

## References

[B1] Schnitker MA, Mattman PE, Bliss TL (1951). A clinical study of malnutrition in Japanese prisoners of war. Ann Intern Med.

[B2] Crook MA, Hally V, Panteli JV (2001). The importance of the refeeding syndrome. Nutrition.

[B3] Hearing S (2004). Refeeding syndrome. BMJ.

[B4] Camp MA, Allon M Severe hypophosphatemia in hospitalised patients. Mineral & Electrolyte Metabolism.

[B5] Marik PE, Bedigan MK Refeeding Hypophosphataemia in an Intensive Care Unit: A Prospective Study. Arch Surg.

[B6] Hayek ME, Eisenberg PG Severe hypophosphatemia following the institution of enteral feedings. Arch Surg.

[B7] McCray S, Walker S, Parrish CR Much ado about refeeding. Practical Gastroenterology.

[B8] Knochel JP The clinical status of hypophosphatemia: an update. NEJM.

[B9] Wacker WEC, Parisi AF Magnesium Metabolism. NEJM.

[B10] Solomon R The relationship between disorders of K+ and Mg+ homeostasis. Semin Nephrol.

[B11] Klein CJ, Stanek GS, Wiles CE Overfeeding macronutrients to critically ill adults. J AmDiet Assoc.

[B12] Reuler JB, Girard DE, Cooney TG Wernicke's encephalopathy. NEJM.

[B13] Veverbrants E, Arky RA Effects of fasting and refeeding: I. Studies on sodium, potassium and water excretion on a constant electrolyte and fluid intake. J Clin Endocrinol Metab.

[B14] National Institute for Health and Clinical Excellence (2006). Nutrition support in adults. Clinical guideline CG32.

[B15] Dewar H, Horvath R, Todorovic VE, Micklewright A (2001). Refeeding syndrome. A pocket guide to clinical nutrition.

